# Integrated Analysis of a Competing Endogenous RNA Network Reveals a Prognostic lncRNA Signature in Bladder Cancer

**DOI:** 10.3389/fonc.2021.684242

**Published:** 2021-08-02

**Authors:** Mou Peng, Xu Cheng, Wei Xiong, Lu Yi, Yinhuai Wang

**Affiliations:** Department of Urology, The Second Xiangya Hospital, Central South University, Changsha, China

**Keywords:** lncRNA signature, ceRNA network, nomogram, prognosis, bladder cancer

## Abstract

Long non-coding RNAs (lncRNAs) act as competing endogenous RNAs (ceRNAs) to regulate mRNA expression through sponging microRNA in tumorigenesis and progression. However, following the discovery of new RNA interaction, the differentially expressed RNAs and ceRNA regulatory network are required to update. Our study comprehensively analyzed the differentially expressed RNA and corresponding ceRNA network and thus constructed a potentially predictive tool for prognosis. “DESeq2” was used to perform differential expression analysis. Two hundred and six differentially expressed (DE) lncRNAs, 222 DE miRNAs, and 2,463 DE mRNAs were found in this study. The lncRNA-mRNA interactions in the miRcode database and the miRNA-mRNA interactions in the starBase, miRcode, and mirTarBase databases were searched, and a competing endogenous RNA (ceRNA) network with 186 nodes and 836 interactions was subsequently constructed. Aberrant expression patterns of lncRNA NR2F1-AS1 and lncRNA AC010168.2 were evaluated in two datasets (GSE89006, GSE31684), and real-time polymerase chain reaction was also performed to validate the expression pattern. Furthermore, we found that these two lncRNAs were independent prognostic biomarkers to generate a prognostic lncRNA signature by univariate and multivariate Cox analyses. According to the lncRNA signature, patients in the high-risk group were associated with a poor prognosis and validated by an external dataset. A novel genomic-clinicopathologic nomogram to improve prognosis prediction of bladder cancer was further plotted and calibrated. Our study deepens the understanding of the regulatory ceRNA network and provides an easy-to-do genomic-clinicopathological nomogram to predict the prognosis in patients with bladder cancer.

## Introduction

Bladder cancer (BC) is the most common urological cancer and the major cause of cancer-related death globally, contributing to 165,100 deaths per year and resulting in significant morbidity and mortality (1). The occurrence and progression of bladder cancer are affected by a variety of intrinsic and extrinsic factors. The most common risk factors include smoking, occupational exposure, and the effects of environmental carcinogens (2). Although a large proportion of bladder cancer is non-muscle invasive bladder cancer (NMIBC) presenting at the first diagnosis, the risk of recurrence is nearly 30%, thus requiring a second transurethral resection of bladder tumor (3). Around 10% to 30% of patients with NMIBC will eventually progress into invasive disease (4). Radical cystectomy after neoadjuvant chemotherapy is a standard treatment for muscle invasive bladder cancer. The 5-year overall survival rate is low (38.6%) among cT3-4aN0 patients after neoadjuvant chemotherapy and radical cystectomy (5). Recently, great progress has been made in the field of immunotherapy by PD-1/PD-L1 inhibitors, which are approved for treating patients with metastatic urothelial carcinoma. However, only one third of the late-stage patients have an initial response to immunotherapy, which may lead to remission (6). The effective options for treating patients with metastatic bladder cancer are extremely limited and required. Therefore, it is of great importance to investigate the molecular mechanisms underlying bladder cancer progression, and thus discover new molecular biomarkers of diagnosis and prognosis for patients with bladder cancer (7).

Long non-coding RNAs (lncRNAs) are a class of RNA defined as transcripts > 200 nt in length, which are transcribed from different regions of the genome (8). Increasing evidence showed that the expression of lncRNAs can not only serve as biomarkers of gene regulation in space, time, developmental stage but also regulate gene expression at the transcriptional, posttranscriptional, and chromosomal levels. lncRNAs are associated with a wide range of biological functions, such as transcriptional regulation, cell growth, and tumorigenesis (9, 10). lncRNA, functioning as competitive endogenous RNA (ceRNA), competes for shared miRNA by cross-regulation among mRNA, circRNA, and pseudogenes (11, 12). This miRNA-regulated lncRNA and mRNA network is a part of the “ceRNA hypothesis” (13). A large number of studies have shown that aberrant expression of lncRNA affects mRNA expression through sponge of miRNA, which is involved in tumorigenesis and cancer progression (8, 14, 15). The development of bladder cancer is accompanied by alterations in the expression patterns of many non-coding RNAs (ncRNAs) (16, 17). Therefore, a better understanding of the regulatory lncRNA network is urgently required for a better functional investigation of the lncRNA.

In this study, we performed an integrated analysis of the transcriptome expression profiles and miRNA-seq data from multiple datasets and identified differentially expressed lncRNA, miRNA, and mRNA to generate a regulatory ceRNA network. We found that lncRNA NR2F1-AS1 and lncRNA AC010168.2 were independent prognostic biomarkers and used for constructing a lncRNA signature to predict the prognosis of bladder cancer. Moreover, a novel genomic-clinicopathological nomogram for predicting survival probability was generated. Our study would help to better understand the regulatory ceRNA network and provide an easy-to-do genomic-clinicopathological nomogram to predict the prognosis in patients with bladder cancer.

## Materials and Methods

### Data Collection and Preprocessing

RNA-seq transcriptomic data and miRNA-seq files were obtained from the TCGA database by using the “GDCRNATools” R package (18). Four hundred thirty-three RNA-seq files and 434 miRNA-seq files were downloaded on June 28, 2019. At the same time, clinical information and survival data (last follow-up time and all causes of death) of 412 patients were downloaded. A total of 403 patients were included in the study by eliminating cases with missing survival information and non-primary bladder cancer. Expression values were normalized by TMM method implemented in edgeR and further transformed by the voom method provided in limma. Low abundance genes (logcpm < 1 in more than half of the samples) were filtered out.

Datasets (GSE89006 and GSE55433) were downloaded from the Gene Expression Omnibus database (https://www.ncbi.nlm.nih.gov/gds/). GSE89006 contained four pairs of adjacent matched bladder tissue and bladder cancer and GSE55433 consisted of 26 bladder tissue samples from healthy controls and 57 bladder cancer samples. GSE89006 and GSE55433 were used as validation cohorts to exhibit the differential expression of lncRNA between bladder cancer and normal bladder tissue. GSE31684, which consisted of 93 bladder cancer patients with survival information, was used as a validation cohort for the prognostic value of lncRNA signature.

### Preparation of *S*amples and Real-Time PCR

Three adjacent bladder tissues and six bladder cancer samples were collected from the Department of Urology, Second Xiangya Hospital of Central South University. Written informed consent was obtained from all patients, and this study was approved by the Clinical Research Ethics Committee of the Second Xiangya Hospital of Central South University. All methods were carried out in accordance with relevant guidelines and regulations (Declaration of Helsinki). Patient samples harvested by the surgical operations were frozen in liquid nitrogen. Real-time PCR was performed as previously described (19). Reverse transcription reaction was performed using Servicebio RT First Strand cDNA Synthesis Kit (G3330, Servicebio, China). Real-time PCR was cycled using 2× SYBR Green qPCR Master Mix (High ROX) (G3322, Servicebio, China). Primers are followed from 5′ to 3′: AC010168.2: CAGAAATGGAGGTGATGTGGC (F), CTCTCAGAGTTCTCAAGGCGTG (R); NR2F1-AS1: GTATTGACAGAGCAGGTAGATGAAAC (F), TTCTATTGCCAAAGCTCCCC(R); GAPDH: GGAAGCTTGTCATCAATGGAAATC (F), TGATGACCCTTTTGGCTCCC (R). This experiment was repeated three times.

### Identification of Differentially Expressed mRNAs, miRNAs, and lncRNAs

Based on CRCh38.90 mapping from ENSEMBLE, mRNA and lncRNA were classified into two different files. Differentially expressed mRNA and lncRNA were analyzed to compare bladder cancer and adjacent bladder tissue by using the R package “DESeq2” with |logFC| ≥2 and FDR < 0.01 as the cutoff value. A volcano plot was used to visualize differentially expressed RNA analysis outcomes. The heatmap was generated by the “ggplot2” package. Regarding those patients with a different risk score, the differentially expressed genes were identified between the low-risk and high-risk groups using R package “limma” with “|logFC| ≥1.5 and FDR < 0.05” as the cutoff value.

### Construction of the lncRNA-miRNA-mRNA ceRNA Network

Differentially expressed lncRNA (DElncRNA), miRNA (DEmiRNA), and mRNA (DEmRNA) were used for further analysis, and lncRNA-miRNA interactions and mRNA-miRNA interactions were analyzed to search for shared miRNAs. As the official description of “GDCRNATools” R package, there are three criteria for determining the ceRNA between lncRNA and mRNA. First, lncRNA and mRNA share a certain number of miRNAs. Second, the correlation between lncRNA and mRNA expression was positive. Third, those shared miRNAs should have similar effects to regulate the expression levels of lncRNA and mRNA (http://bioconductor.org/packages/devel/bioc/vignettes/GDCRNATools/inst/doc/GDCRNATools.html#competing-endogenous-rnas-network-analysis). The lncRNA-miRNA pairs were investigated by miRcode (20) database, and the miRNA-mRNA pairs were used to identify three databases: starBase v2.0 (21), miRcode (20), and miRTarBase v7.0 (22). The Pearson correlation coefficient was evaluated to assess the linear association between lncRNA and mRNA. The included criteria were as follows: p-value of hypergeometric test and correlation was less than 0.01, and the absolute value of correlation was more than 0.4. Modulation of similarity and sensitivity correlations was used to calculate the possible regulatory effects of consensus miRNAs on lncRNA and mRNA. The function of “gdcExportNetwork” in this package was used to analyze the lncRNA-miRNA-mRNA interactions and the “edges” and “nodes” files were sequentially generated. Only the overlapped miRNA-mRNA interactions in these three databases were retained and the “edges” and “nodes” files were updated. Subsequently, all files were imported into Cytoscape (version 3.6.1) for the visualization of ceRNA network.

### Functional Enrichment Analysis

Gene ontology (GO) and Kyoto Encyclopedia of Genes and Genomes (KEGG) pathways were analyzed to investigate the function of differentially expressed mRNAs in the ceRNA network based on the Database for Annotation, Visualization, and Integrated Discovery (DAVID) (version 6.8, https://david.ncifcrf.gov/) (23). Differentially expressed genes between the low-risk and the high-risk groups were analyzed using R packages “clusterProfiler” (24) and “DOSE” (25). These mRNAs were enriched in molecular function, cellular component, biological process, and signaling pathways, which was considered significant when p<0.05.

### PPI Network Analysis for Protein-Protein Interactions

Differentially expressed mRNAs involved in the ceRNA network were subjected to the searching tool for the retrieval of interacting genes (STRING) for protein-protein interaction network analysis (version 11.0, https://string-db.org/) (26). The database scored to predict the interactions of proteins. All settings were the default values. TSV file was downloaded, and then PPI network was generated by Cytoscape according to the PPI correlations. This PPI network was further analyzed to search clusters by an MCODE plug-in in the Cytoscape. Advanced options were as follows: degree cutoff: 2, haircut, nodes score cutoff: 0.2, K-core: 2, Max. Depth: 100.

### Construction of lncRNA *S*ignature Based on the ceRNA Network

Univariate Cox proportional hazards regression analysis was performed to screen differentially expressed lncRNAs, which were associated with overall survival. Differentially expressed lncRNAs were selected for further analysis when p-value was less than 0.05. Multivariate Cox proportional hazards regression analysis was subsequently performed, and lncRNAs were considered as the significant difference when p value was less than 0.1. Based on these independent prognostic lncRNAs, the risk score of each patient with bladder cancer was calculated. The calculation formula of risk score equals to coef (LNC1) *exp (LNC1) +coef (LNC2) *exp (LNC2) +…+coef (LNCn)*exp (LNCn), in which coef (LNC) is a coefficient obtained by calculation of multivariate Cox analysis and exp (LNC) is normalized expression value. The median risk score was set as a cutoff, and patients were divided into two groups: low-risk group and high-risk group. Kaplan-Meier curve was plotted to predict the overall survival between the low-risk and high-risk groups. ROC curve was analyzed to evaluate the predictive power of this lncRNA signature for overall survival.

### Gene Set Enrichment Analysis

According to patients with a risk score, Gene Set Enrichment Analysis (GSEA) (version 4.0.3, https://www.gsea-msigdb.org/gsea/index.jsp) was performed to determine DEGs that were enriched in gene lists extracted from MSigDB (27). KEGG pathways that were enriched were determined by gene sets from the curated C2,cp.kegg.v7.0.symbols.gmt collections. The number of permutations was 1,000.

### Estimate

Based on the expression data of TCGA, the level of stromal cells was presented, and the infiltration levels of immune cells in bladder cancer were calculated and downloaded from Estimation of STromal and Immune cells in MAlignant Tumor tissues using Expression data (ESTIMATE) (28) (version 1.0.13, https://bioinformatics.mdanderson.org/estimate/). The differences in the stromal score and the immune score were analyzed between the low-risk and high-risk groups.

### Clinical Application of lncRNA *S*ignature

According to clinical characteristics, stratified survival analysis was performed, and Kaplan-Meier curves of clinical characteristics were plotted. Then, univariate and multivariate Cox regression proportional hazards regression analyses were performed to find independent prognostic factors of bladder cancer. In addition, a novel nomogram to predict the possibility of 1,3,5-year survival was established after stepwise regression analysis according to clinical characteristics by the package of rms (29). The nomogram was composed of four components: points line, risk factors line, survival probability line, and total points line. The length of each risk factor line reflects the regression coefficient estimated by multiple logistic regression analyses. The calibration was performed to evaluate the predictive value of the nomogram. To our knowledge, a calibration plot is drawn to verify the degree of calibration and an ideal line in the calibration plot is a 45-degree angle. The proposed nomogram has an accurate predictive ability when the actual graph is overlapped with the ideal line. All analyses were performed using R studio software version 1.2.5019 and R software version 3.6.2.

## Results

### Identification of Differentially Expressed lncRNA, miRNA, and mRNA

**Figure 1:**
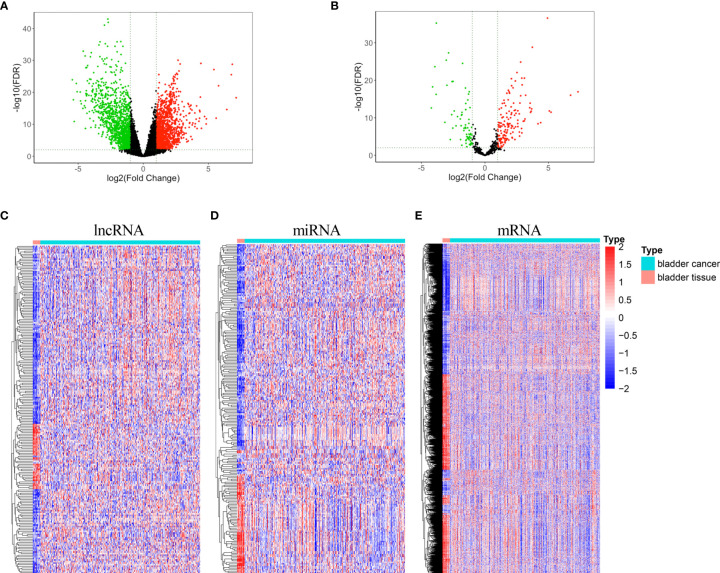
Volcano plots and heatmaps of differentially expressed lncRNA, miRNA, and mRNA. **(A)** Volcano plot of differentially expressed mRNA and lncRNA **(B)** Volcano plot of differentially expressed miRNA. **(C)** Heatmap of differentially expressed lncRNA. **(D)** Heatmap of differentially expressed miRNA. **(E)** Heatmap of differentially expressed mRNA.

Two hundred and six differentially expressed lncRNA, 222 differentially expressed miRNA, and 2,463 differentially expressed mRNA were obtained by “DESeq2” analysis with cutoff (|logFC| ≥2 and p < 0.01). The volcano plot of mRNA and lncRNA was shown in **Figure 1A**, and the volcano plot of miRNA was displayed in **Figure 1B**. Heatmaps of lncRNA, miRNA, and mRNA are shown in **Figures 1C–E**, respectively. The clinical characteristics of patients with bladder cancer, including age, gender, race, pathologic stage, pathologic T, pathologic N, pathologic M, and vital status, were listed in **Table S1**.

### Construction of a lncRNA-miRNA-mRNA ceRNA Network

**Figure 2:**
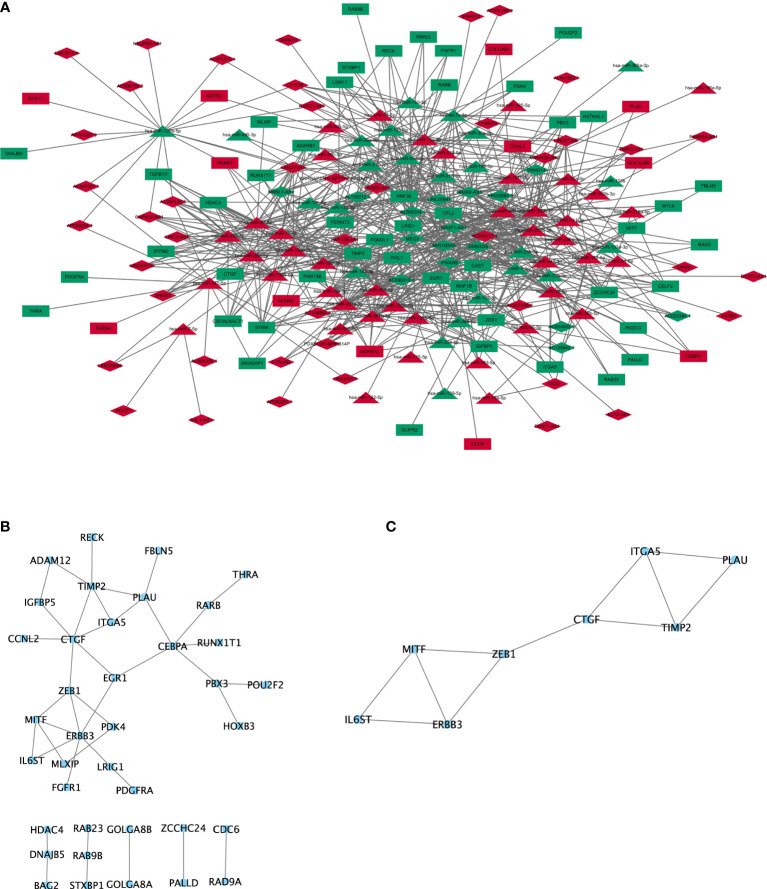
ceRNA network and protein-protein interaction network. **(A)** lncRNA-miRNA-mRNA ceRNA network generated by Cytoscape (version 3.6.1, https://cytoscape.org/). Red: upregulated RNAs. Green: downregulated RNAs. Triangle: miRNA. Diamond: lncRNA. Rectangle: mRNA. **(B)** PPI network of mRNA involved in the ceRNA network were generated by STRING (version 11.0, https://string-db.org/). **(C)** Subnetwork of PPI cluster.

Based on miRcode database for lncRNA-miRNA interactions and starBase, miRcode, and mirTarBase databases for mRNA-miRNA interactions, 60 DElncRNA (44 upregulated lncRNA and 16 downregulated lncRNA), 66 DEmiRNA (42 upregulated miRNAs and 24 downregulated miRNA), and 60 DEmRNA (12 upregulated mRNA and 48 downregulated mRNA) were obtained as nodes, and 836 interactions were displayed as edges. A lncRNA-miRNA-mRNA ceRNA network was subsequently constructed by “Cytoscape” (**Figure 2A**). In this ceRNA network, we found that TIMP2 was a potential target of hsa-miR-200b/c-3p, serving as a natural inhibitor of the matrix metalloproteinases and a potential non-invasive biomarker in kidney cancer and lung cancer. NR2F1-AS1 was also a potential target of hsa-miR-200b/c-3p and may act as a ceRNA in regulating the expression level of TIMP2 through competitive binding to hsa-miR-200b/c-3p.

### GO and KEGG Pathway Analysis Revealed That mRNAs in the ceRNA Network Were Enriched in Cancer-Associated Signaling Pathways

The results from DAVID online tool (23) revealed that mRNAs in the ceRNA network were enriched in biological process, including “negative regulation of transcription from RNA polymerase II promoter”, “cartilage development”, “positive regulation of phospholipase C activity”, “transcription, DNA-templated”, “lung development”, “extracellular matrix organization”, “integrin-mediated signaling pathway”, and “xenophagy”. The enriched cellular components were involved in “nucleus”. The enriched molecular functions were involved in “sequence-specific DNA binding”, “DNA-binding transcription factor activity”, and “transcription corepressor activity”. The enriched KEGG signaling pathways were involved in “microRNAs in cancer”, “regulation of actin cytoskeleton”, “pathways in cancer”, “transcriptional misregulation in cancer”, and “melanoma” (**Table S2**).

### PPI Network Indicated That mRNAs in the ceRNA Network Were Further Clustered in Cancer-Associated Signaling Pathways

The protein-protein interactions network (26) was reconstructed by Cytoscape, and protein-coding mRNAs in the ceRNA network showed strong interactions (**Figure 2B**). To further investigate clusters (highly interconnected regions), plug-in of MCODE was used in Cytoscape, and eight proteins (IL6ST, ERBB3, MITF, ZEB1, CTGF, TIMP2, ITGA5, and PLAU) were obtained in one cluster (**Figure 2C**). Four proteins (PLAU, ITGA5, ZEB1, and ERBB3) were involved in “microRNAs in cancer” signaling pathway. Three proteins (PLAU, ZEB1, and MITF) were involved in the “transcriptional misregulation in cancer” (**Figure 2C**).

### Validation of Differentially Expressed lncRNAs of NR2F1-AS1 and AC010168.2 as Independent Prognostic Biomarkers in Bladder Cancer

**Figure 3:**
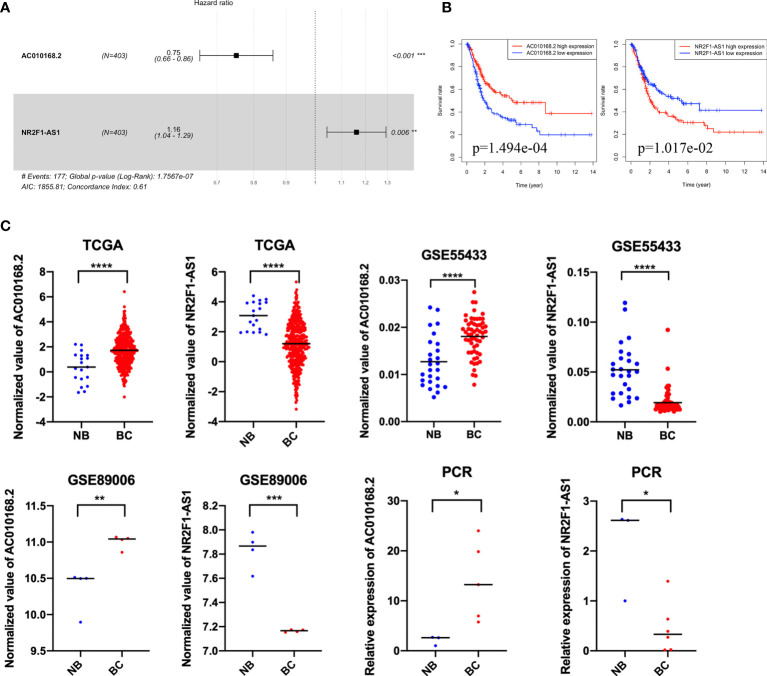
The expression pattern and prognostic value of AC010168.2 and NR2F1-AS1. **(A)** Forest plot of AC010168.2 and NR2F1-AS1 was involved in the ceRNA network. **(B)** Kaplan-Meier curve of AC010168.2 and NR2F1-AS1. **(C)** The expression of AC010168.2 and NR2F1-AS1 in bladder cancer tissue and adjacent normal tissue. Validation of cohorts (GSE89006 and GSE55433). These results were also validated by real-time PCR. *p < 0.05, **p < 0.01, ***p < 0.001, ****p < 0.0001.

Among these differentially expressed lncRNAs in this ceRNA network, 27 lncRNAs were associated with the prognosis of overall survival through univariate Cox regression (p<0.05) (**Table S3**). Subsequently, multivariate Cox analysis was performed for these 27 lncRNAs. Finally, two lncRNAs (NR2F1-AS1 and AC010168.2) were obtained for the construction of a prognostic lncRNA signature (**Figure 3A**) (**Table S4**). High expression of NR2F1-AS1 was associated with a poorer prognosis (HR, 1.16; p = 1.017e-02), whereas high expression of AC010168.2 was associated with a better prognosis (HR, 0.75; p = 1.494e-04) (**Figure 3B**). Training datasets (TCGA-BLCA) and validation datasets (GSE89006, GSE31684) indicated that AC010168.2 expression was elevated in the high-risk group, whereas NR2F1-AS1 expression was decreased in the high-risk group, this was further validated by real-time PCR (**Figures 3C** and **S1**). The clinical information of training and validation cohorts are shown in **Table 1**.

**Table 1:** Training cohort and validation cohorts.

	Training cohort (TCGA-BLCA)		Validation cohort 1 (GSE55433)		Validation cohort 2 (GSE31684)
	Urothelium Carcinoma	Normal Urothelium	Urothelium Carcinoma	Normal Urothelium	Urothelium Carcinoma
Total	403	19	57	26	93
Gender					
Male	297	10	39	6	68
Female	106	9	12	1	25
Age (year)					
Mean	68	69.9	72.4	65.5	69.1
Range	34-90	48-90	46-89	59-82	42-91
Stage					
pTa	0		26		5
pTis	0		2		
pT1	2		9		10
pT2-T4	399		19		78
pT2	129				17
pT3	138				42
pT4	132				19

### Identification of a 2-lncRNA Prognostic Signature Based on the ceRNA Network

**Figure 4:**
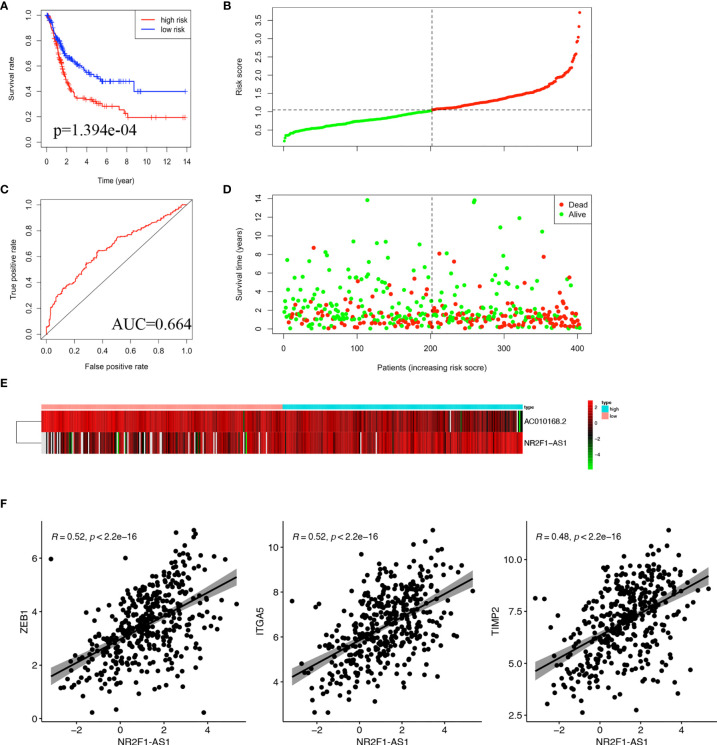
Construction of lncRNA signature in TCGA-BLCA cohort. **(A)** Kaplan-Meier curve was plotted for overall survival of bladder cancer patients. **(B)** ROC curve in the high-risk and low-risk groups based on 2-lncRNA signature. **(C)** Risk score distribution. **(D)** Survival status distribution. **(E)** Heatmap of 2 lncRNA in the prognostic model. **(F)** Pearson correlation analysis between NR2F1-AS1 and ZEB1, ITGA5, TIMP2.

Based on the coefficient of NR2F1-AS1 and AC010168.2 of multivariate Cox analysis, a prognostic lncRNA signature was constructed. The formula of this signature was as follows: risk score= −0.2863566*exp (AC010168.2) + 0.1498769*exp (NR2F1-AS1). The lncRNA signature was associated with poorer overall survival in the high-risk group (p = 1.394e-04) (**Figure 4A**). To investigate the sensitivity and specificity of this prognostic risk model, the ROC curve was plotted, and the area under curve (AUC) was 0.664 (**Figure 4B**). Based on the median risk score as cutoff, 403 bladder cancer patients were divided into two groups (**Figure 4C**), and more deaths occurred in the high-risk group (**Figure 4D**). The expression level of NR2F1-AS1 was gradually elevated in pace with an increased risk score, but the expression level of AC010168.2 was decreased (**Figure 4E**).

To further analyze the function of these two lncRNAs, Pearson correlation analysis was performed between lncRNA and mRNA. There were significant correlations in pairs of NR2F1-AS1/ZEB1 (cor = 0.52, p<2.2e-16), NR2F1-AS1/ITGA5 (cor=0.52, p < 2.2e-16), and NR2F1-AS1/TIMP2 (cor=0.48, p<2.2e-16), respectively (**Figure 4F**). It was suggested that there were multiple miRNAs-regulated NR2F1-AS1 that affected the expression levels of ZEB1, ITGA5, and TIMP2.

### The lncRNA Signature Was Possibly Related to ECM Receptor Interaction in the Tumor Microenvironment

**Figure 5:**
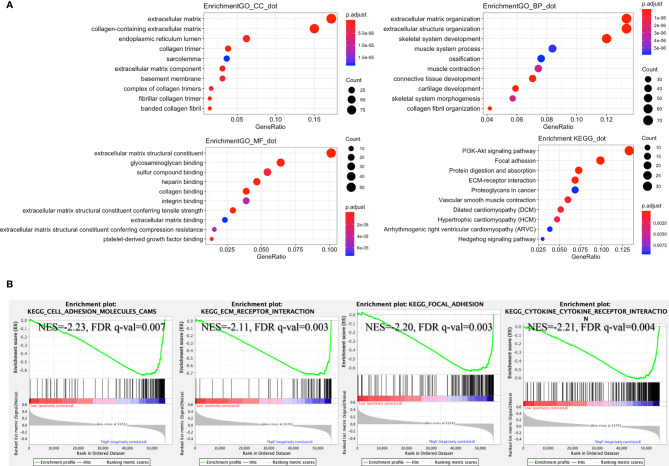
Enrichment of signaling pathways in the high-risk group. **(A)** According to the risk score, GO and KEGG signaling pathways were explored by R packages “clusterProfiler” and “DOSE.” **(B)** Gene set enrichment analysis (GSEA) indicated that “cell adhesion molecules cams”, “ECM receptor interaction”, “focal adhesion”, and “cytokine-cytokine receptor interaction” were enriched in the high-risk group (GSEA version 4.0.3, https://www.gsea-msigdb.org/gsea/index.jsp).

According to the lncRNA signature, risk-associated differentially expressed genes were analyzed to investigate GO function and KEGG signaling pathways. Five hundred seventy-two differentially expressed genes were obtained and used for further analysis. The main aspect of GO enrichment was related to the extracellular matrix. KEGG pathways enrichment analysis showed that lncRNA signature was involved in PI3K-Akt signaling pathway, focal adhesion, and ECM-receptor interaction (**Figure 5A**). Furthermore, GSEA indicated that ECM-receptor interaction, cell adhesion molecules cams, focal adhesion and cytokine-cytokine receptor interaction were enriched in the high-risk group (**Figure 5B**). Based on the heatmap of characterized signatures, our risk model could separate luminal (low risk) and basal (high risk) subtypes. Most of the signatures of ECM and smooth muscle, EMT and Claudin markers were displayed in the high-risk group (**Figure S2**).

### The lncRNA Signature Was Related to Cancer-Associated Fibroblast in Bladder Cancer

**Figure 6:**
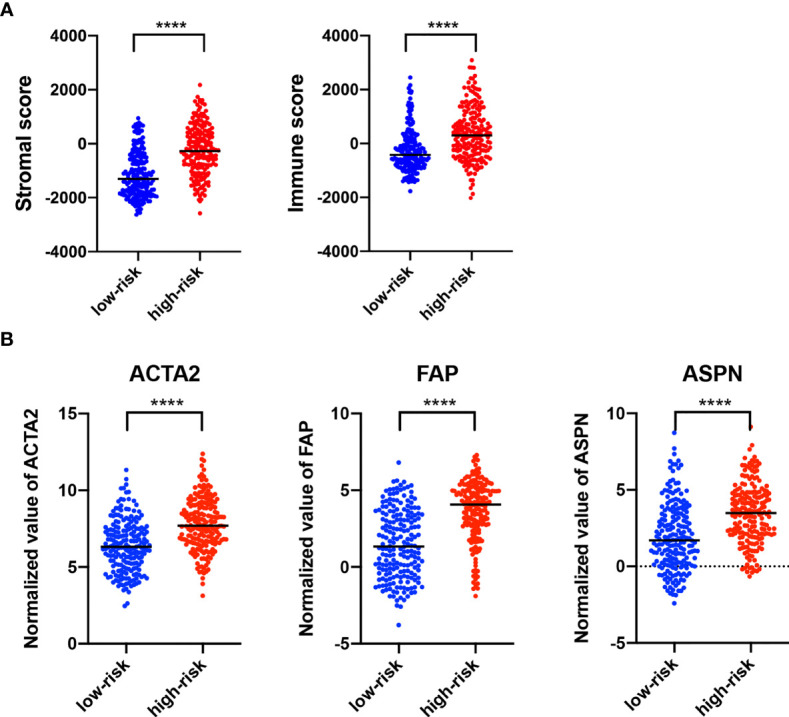
Characterization of Stromal and immune infiltration based on the lncRNA signature. **(A)** ESTIMATE analysis (version 1.0.13, https://bioinformatics.mdanderson.org/estimate/) of stromal score and immune score. **(B)** Expression of different biomarkers in cancer-associated fibroblasts in the high-risk group as compared with the low-risk group. ****p < 0.0001.

Estimation of STromal and Immune cells in MAlignant Tumor tissues using Expression data (ESTIMATE) analysis revealed that stromal score and immune score were both higher in the high-risk group than in the low-risk group (**Figure 6A**). The tumor microenvironment is a complex mixture of tumor cells in the extracellular matrix (ECM), including diffusible growth factors, cytokines, and various types of stromal cells. It is believed that cancer-related fibroblasts (CAFs) are actively involved in the metastasis and invasion of tumor cells and CAFs are closely related to the poor prognosis of a variety of cancers (30). All CAFs expressed certain mesenchymal markers, including α-SMA, vimentin, and FAP. We found that the expression of ACTA2 (gene coded α-SMA), FAP, and ASPN were upregulated in the high-risk group, as compared to the low-risk group (**Figure 6B**).

### Validation of Prognostic Value of lncRNA Signature and Correlation Between lncRNA Signature and Biomarkers of Cancer-Associated Fibroblasts

**Figure 7:**
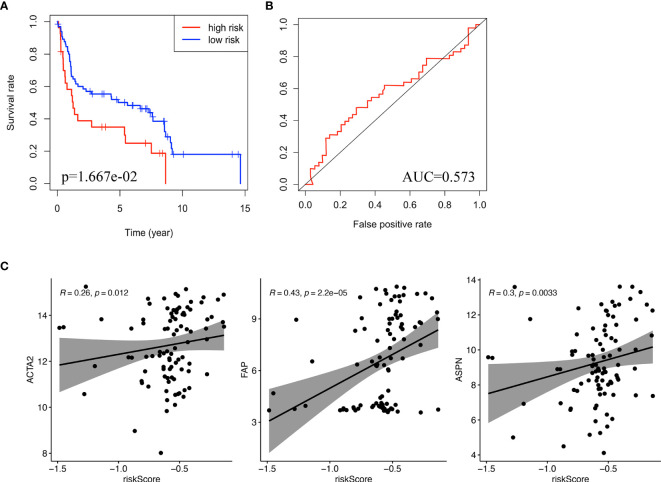
Validation of the lncRNA signature. **(A)** Kaplan-Meier curve for overall survival of bladder cancer patients in validation cohort (GSE31486). **(B)** ROC curve and AUC of the lncRNA signature in validation cohort. **(C)** The correlation between risk score and biomarkers of fibroblasts (ACTA2, FAP, ASPN) in validation cohort. ROC, receiver operating characteristic; AUC, area under the ROC curve.

GSE31684 consists of 93 bladder cancer patients managed by radical cystectomy (31). The detailed clinical information of GSE31684 is shown in **Table 1**. The survival information of patients and expression values of NR2F1-AS1, AC010168.2, ACTA2, FAP, and ASPN were extracted. According to the correlation coefficient, the lncRNA signature in GSE31684 was recalculated, and Kaplan-Meier curve of overall survival was displayed, indicating that the high-risk subgroup was associated with a poor prognosis (p = 1.667e-2) (**Figure 7A**). The AUC was 0.573 (**Figure 7B**). Risk score of lncRNA signature was positively correlated with ACTA2 (R=0.26, p=0.012), FAP (R=0.43, p=2.2e-05) and ASPN (R=0.30, p=0.0033) (**Figure 7C**).

### The Prognostic lncRNA Signature Is an Independent Prognostic Factor in Bladder Cancer

**Figure 8:**
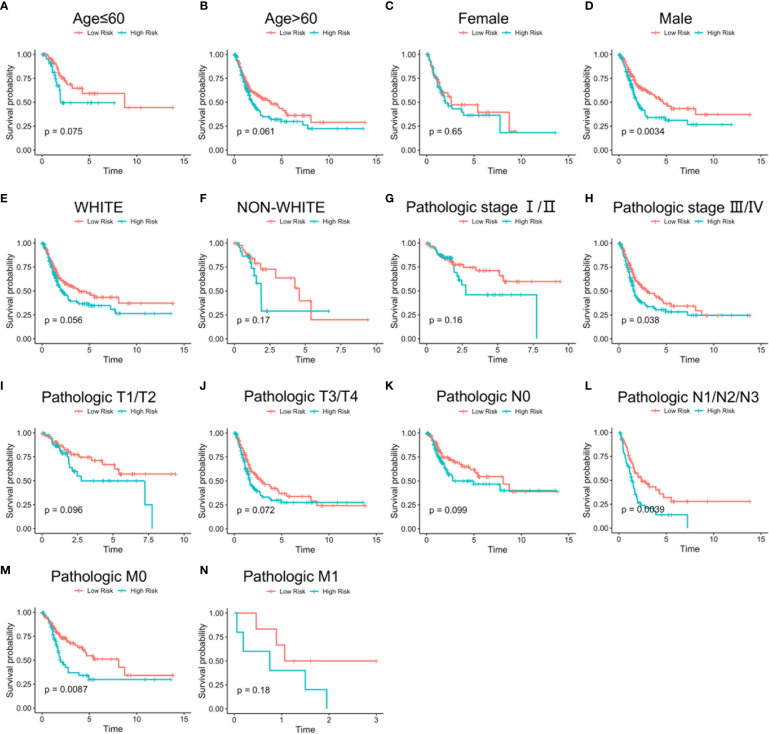
Stratified survival analysis based on clinical characteristics. Overall survival analysis of age ≤ 60 **(A)**, age > 60 **(B)**, female **(C)**, male **(D)**, White **(E)**, non-White **(F)**, pathologic stage I/II **(G)**, pathologic stage III/IV **(H)**, pathologic T1/T2 **(I)**, pathologic T3/T4 **(J)**, pathologic N0 **(K)**, pathologic N1/N2/N3 **(L)**, pathologic M0 **(M)**, and pathologic M1 **(N)** patients according to risk score. Time: Years.

**Figure 9:**
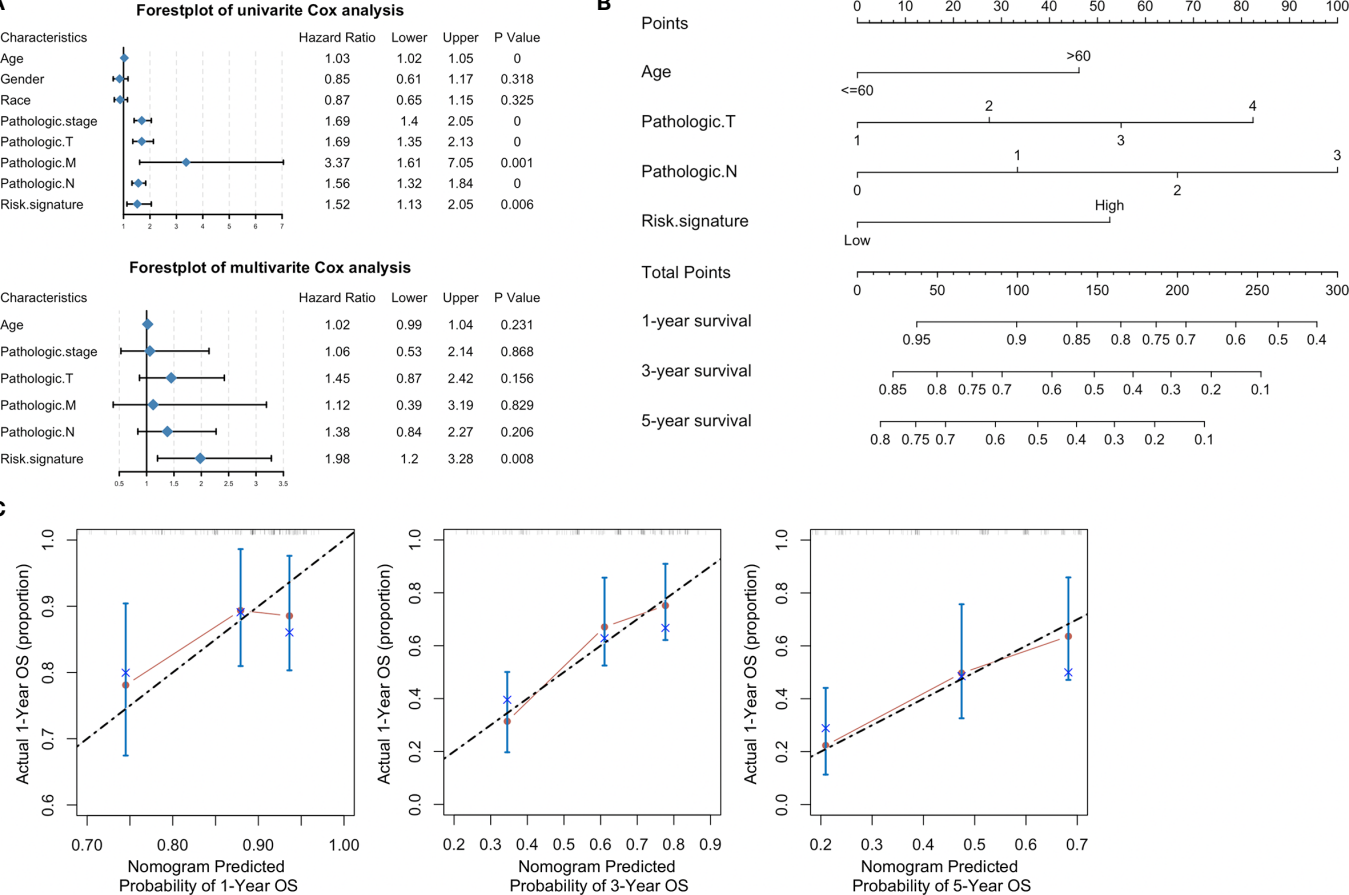
A nomogram based on clinical characteristics. **(A)** Forest plot of clinical characteristics after univariate and multivariate Cox analyses. **(B)** Nomogram of prediction for 1-, 3-, 5-year survivals. **(C)** Calibration curves of the nomogram-predicted 1-, 3-, 5-year survivals.

Stratified analysis of overall survival indicated that the high-risk score was associated with a poor prognosis in the subgroups of gender (male), pathologic stage (III-IV), pathologic N (N1-N3), pathologic M (M0) according to lncRNA signature (**Figure 8**). Subsequently, univariate Cox analysis was performed. Age, pathologic stage, pathologic T, pathologic M, pathologic N, and lncRNA signature were significantly different and associated with prognosis. Multivariate Cox analysis revealed that only the lncRNA signature was an independent prognostic factor in bladder cancer (**Figure 9A**).

### A Novel Nomogram Was Generated for Clinical Application to Predict Overall Survival of Patients With Bladder Cancer

Based on the clinical characteristics of bladder cancer, we found four clinical characteristics (age, pathologic T, pathologic N, and risk signature) after stepwise Cox analysis for the establishment of a novel genomic-clinicopathological nomogram to predict a 1-, 3-, 5-year survivals (**Figure 9B**). The mortality score for each patient was calculated based on the scoring system at the bottom of the nomogram. A calibration of 1-, 3-, 5-year survival curves based on self-service resampling verification was consistent with nomogram predictions and was observed survival possibility (**Figure 9C**).

## Discussion

Accurate predictions of overall survival could be extremely beneficial for patients with bladder cancer. Recently, many studies found varieties of diagnostic and prognostic biomarkers for cancer (32–34), few of which were utilized in clinical administration. A good biomarker can help identify cancers in the early stage, risk stratification, prognosis improvement, outcome prediction, variant histology (35), and targeted therapy (36). In this context, it is important to identify biomarkers that enable accurate prediction of recurrence, progression, and clinical outcome in bladder cancer. Thus, it is of great interest that we found prognostic lncRNA biomarkers and generated a lncRNA signature to evaluate 1-, 3-, 5-year overall survivals in bladder cancer. A genomic-clinicopathological nomogram was established, and this could help physicians to predict the overall survival of patients with bladder cancer.

lncRNA is a type of novel biomarker for clinical procedures involving the diagnosis and prognosis of bladder cancer (37). In many studies, lncRNA is also considered a potential target for cancer treatment (38). Although it was found that some lncRNAs were associated with a good prognosis in bladder cancer, the role and clinical significance of lncRNAs in bladder cancer remained largely unknown. The hypothesis is that ceRNA provides a new approach to study lncRNAs. ceRNA was studied in many biological processes. It has been shown that dysregulated ceRNA networks are closely related to tumorigenesis (39). lncRNAs can sponge with miRNAs to reduce the interaction of miRNA with mRNA. The ceRNA hypothesis provides a better understanding of the relationship among lncRNA, miRNA, and mRNA. In our study, we obtained 60 DElncRNA, 66 DEmiRNA, and 60 DEmRNA for generating a regulatory network of ceRNA. This ceRNA network could, thus, provide a new perspective to investigate the function of lncRNA.

Based on the ceRNA network, two lncRNAs (NR2F1-AS1 and AC010168.2) were found and served as prognostic biomarkers. Previous studies have been shown that the expression of AC010168.2, also called RP11-174G6.5, may differ between the normal bladder and para-carcinoma tissue (40). More studies are focusing on NR2F1-AS1, which promotes the proliferation and migration in thyroid cancer by regulating the miRNA-338/CCND1 axis (41). NR2F1-AS1 targets SOX1 *via* sponging miR-363 to regulate the progression of endometrial cancer (42) and induces oxaliplatin resistance through miR-363 to target ABCC1 in hepatocellular carcinoma (43). We found that NR2F1-AS1 was positively correlated with ZEB1, ITGA5 (integrin subunit gene), and TIMP2 (gene of natural inhibitors of the matrix metalloproteinases), respectively. As the principal component of lncRNA signature, NR2F1-AS1 may regulate the integrin and degradation of extracellular matrix. It is suggested that lncRNA signature may play an important role in the tumor microenvironment through extracellular matrix, which is secreted by cancer-associated fibroblasts in bladder cancer. The results from KEGG pathways and GSEA also suggested a strong correlation between lncRNA signature and extracellular matrix. Furthermore, there is a cross-talk among TGF-β signaling pathways, integrins, and the extracellular matrix (44). TGF-β–associated extracellular matrix genes link cancer-associated fibroblasts to immune evasion and immunotherapy failure (45). TGF-β blockade enhances specific responses to the immune-checkpoint blockade (46), which further provides an evidence that patients with this lncRNA signature could benefit from a potential treatment of TGF-β blockade. Higher immune score and stromal score in the high-risk group implied therapeutic potential of combination treatments of immune checkpoint inhibitor and TGF-β blockade in metastatic bladder cancer.

Cancer-specific survival was associated with a variety of clinical features, including age, higher tumor grade, higher pathological stage, lymph node metastasis, lymphovascular invasion, and soft tissue surgical margin (47). According to the lncRNA signature, a stratified analysis of overall survival indicated that a high-risk score was associated with a poor prognosis in the subgroups of gender (male), pathologic stage (III-IV), pathologic N (N1-N3), pathologic M (M0). The lncRNA signature is an independent prognostic factor in bladder cancer. Furthermore, different from other nomograms (48, 49), we generated a novel genomic-clinicopathological nomogram with four variables to predict mortality of patients with bladder cancer. The nomogram in this study may be a novel useful tool for clinical application.

There were some limitations in this study. First, further experiments were required for verifying the role of AC010168.2 and NR2F1-AS1 in affecting the prognosis of patients with bladder cancer. Second, following with more discovered interactions among lncRNA, miRNA, and mRNA, the lncRNA-miRNA-mRNA regulatory ceRNA network is dynamically updated.

In conclusion, we constructed a regulatory lncRNA-miRNA-mRNA ceRNA network and generated a novel genomic-clinicopathological nomogram to predict survival rate. Our study may deepen a novel understanding of a regulatory ceRNA network and provide an easy-to-do genomic-clinicopathological nomogram to predict the prognosis of patients with bladder cancer.

## Data Availability Statement

The datasets presented in this study can be found in online repositories. The names of the repository/repositories and accession number(s) can be found in the article/**Supplementary Material**.

## Ethics Statement

The studies involving human participants were reviewed and approved by Clinical Research Ethics Committee of the Second Xiangya Hospital of Central South University. The patients/participants provided their written informed consent to participate in this study.

## Author Contributions

MP and YW designed the project. MP and XC acquired data and wrote the manuscript. LY and YW performed surgical operations to obtain bladder cancer samples. WX performed real-time PCR. WX, LY, YW, and MP reviewed the manuscript. MP and YW supervised the study. All authors contributed to the article and approved the submitted version.

## Conflict of Interest

The authors declare that the research was conducted in the absence of any commercial or financial relationships that could be construed as a potential conflict of interest.

## Publisher’s Note

All claims expressed in this article are solely those of the authors and do not necessarily represent those of their affiliated organizations, or those of the publisher, the editors and the reviewers. Any product that may be evaluated in this article, or claim that may be made by its manufacturer, is not guaranteed or endorsed by the publisher.

## References

[1] Chen W Zheng R Baade PD Zhang S Zeng H Bray F. Cancer Statistics in China, 2015. CA Cancer J Clin (2016) 66(2):115–32. 10.3322/caac.21338 26808342

[2] Cumberbatch MG Cox A Teare D Catto JW. Contemporary Occupational Carcinogen Exposure and Bladder Cancer: A Systematic Review and Meta-Analysis. JAMA Oncol (2015) 1(9):1282–90. 10.1001/jamaoncol.2015.3209 26448641

[3] Felsenstein KM Theodorescu D. Precision Medicine for Urothelial Bladder Cancer: Update on Tumour Genomics and Immunotherapy. Nat Rev Urol (2018) 15(2):92–111. 10.1038/nrurol.2017.179 29133939

[4] Santoni G Morelli MB Amantini C Battelli N. Urinary Markers in Bladder Cancer: An Update. Front Oncol (2018) 8:362. 10.3389/fonc.2018.00362 30245975 PMC6137202

[5] Nitta M Kuroda S Nagao K Higure T Zakoji H Miyakita H. Effect of Neoadjuvant Chemotherapy in Patients Undergoing Radical Cystectomy for Muscle-Invasive Bladder Cancer: A Retrospective, Multi-Institutional Study. Jpn J Clin Oncol (2019) 50(1):73–9. 10.1093/jjco/hyz137 31612911

[6] Pichler R Horninger W Heidegger I. Asco 2018: Highlights of Urothelial Cancer and Prostate Cancer. Memo (2018) 11(4):284–90. 10.1007/s12254-018-0422-0 PMC6280775 30595755

[7] Sanguedolce F Russo D Mancini V Selvaggio O Calo B Carrieri G. Morphological and Immunohistochemical Biomarkers in Distinguishing Prostate Carcinoma and Urothelial Carcinoma: A Comprehensive Review. Int J Surg Pathol (2019) 27(2):120–33. 10.1177/1066896918814198 30509113

[8] Schmitt AM Chang HY. Long Noncoding RNAs in Cancer Pathways. Cancer Cell (2016) 29(4):452–63. 10.1016/j.ccell.2016.03.010 PMC4831138 27070700

[9] Kornienko AE Guenzl PM Barlow DP Pauler FM. Gene Regulation by the Act of Long Non-Coding RNA Transcription. BMC Biol (2013) 11:59. 10.1186/1741-7007-11-59 23721193 PMC3668284

[10] Fatica A Bozzoni I. Long Non-Coding RNAs: New Players in Cell Differentiation and Development. Nat Rev Genet (2014) 15(1):7–21. 10.1038/nrg3606 24296535

[11] Gregory PA Bert AG Paterson EL Barry SC Tsykin A Farshid G. The miR-200 Family and miR-205 Regulate Epithelial to Mesenchymal Transition by Targeting ZEB1 and SIP1. Nat Cell Biol (2008) 10(5):593–601. 10.1038/ncb1722 18376396

[12] Zhang N Wang X Huo Q Sun M Cai C Liu Z. MicroRNA-30a Suppresses Breast Tumor Growth and Metastasis by Targeting Metadherin. Oncogene (2014) 33(24):3119–28. 10.1038/onc.2013.286 23851509

[13] Thomson DW Dinger ME. Endogenous MicroRNA Sponges: Evidence and Controversy. Nat Rev Genet (2016) 17(5):272–83. 10.1038/nrg.2016.20 27040487

[14] Zhang A Zhang J Kaipainen A Lucas JM Yang H. Long Non-Coding RNA: A Newly Deciphered “Code” in Prostate Cancer. Cancer Lett (2016) 375(2):323–30. 10.1016/j.canlet.2016.03.003 26965999

[15] Xu Y Jiang X Cui Y. Upregulated Long Noncoding RNA Pandar Predicts an Unfavorable Prognosis and Promotes Tumorigenesis in Cholangiocarcinoma. Onco Targets Ther (2017) 10:2873–83. 10.2147/OTT.S137044 PMC5476724 28652769

[16] Dyrskjot L Ostenfeld MS Bramsen JB Silahtaroglu AN Lamy P Ramanathan R. Genomic Profiling of microRNAs in Bladder Cancer: miR-129 Is Associated With Poor Outcome and Promotes Cell Death *In Vitro* . Cancer Res (2009) 69(11):4851–60. 10.1158/0008-5472.CAN-08-4043 19487295

[17] Peter S Borkowska E Drayton RM Rakhit CP Noon A Chen W. Identification of Differentially Expressed Long Noncoding RNAs in Bladder Cancer. Clin Cancer Res (2014) 20(20):5311–21. 10.1158/1078-0432.CCR-14-0706 25165097

[18] Li R Qu H Wang S Wei J Zhang L Ma R. GDCRNATOOLs: An R/Bioconductor Package for Integrative Analysis of lncRNA, miRNA and mRNA Data in GDC. Bioinformatics (2018) 34(14):2515–7. 10.1093/bioinformatics/bty124 29509844

[19] Wang Z Wang Y Peng M Yi L. UBASH3B Is a Novel Prognostic Biomarker and Correlated With Immune Infiltrates in Prostate Cancer. Front Oncol (2019) 9:1517. 10.3389/fonc.2019.01517 32010618 PMC6974685

[20] Jeggari A Marks DS Larsson E. Mircode: A Map of Putative Microrna Target Sites in the Long Non-Coding Transcriptome. Bioinformatics (2012) 28(15):2062–3. 10.1093/bioinformatics/bts344 PMC3400968 22718787

[21] Li JH Liu S Zhou H Qu LH Yang JH. starBase V2.0: Decoding miRNA-ceRNA, miRNA-ncRNA and Protein-RNA Interaction Networks From Large-Scale CLIP-Seq Data. Nucleic Acids Res (2014) 42(Database issue):D92–7. 10.1093/nar/gkt1248 PMC3964941 24297251

[22] Chou CH Shrestha S Yang CD Chang NW Lin YL Liao KW. Mirtarbase Update 2018: A Resource for Experimentally Validated microRNA-target Interactions. Nucleic Acids Res (2018) 46(D1):D296–302. 10.1093/nar/gkx1067 PMC5753222 29126174

[23] Huang da W Sherman BT Lempicki RA. Systematic and Integrative Analysis of Large Gene Lists Using DAVID Bioinformatics Resources. Nat Protoc (2009) 4(1):44–57. 10.1038/nprot.2008.211 19131956

[24] Yu G Wang LG Han Y He QY. clusterProfiler: An R Package for Comparing Biological Themes Among Gene Clusters. OMICS (2012) 16(5):284–7. 10.1089/omi.2011.0118 PMC3339379 22455463

[25] Yu G Wang LG Yan GR He QY. Dose: An R/Bioconductor Package for Disease Ontology Semantic and Enrichment Analysis. Bioinformatics (2015) 31(4):608–9. 10.1093/bioinformatics/btu684 25677125

[26] Szklarczyk D Gable AL Lyon D Junge A Wyder S Huerta-Cepas J. String V11: Protein-Protein Association Networks With Increased Coverage, Supporting Functional Discovery in Genome-Wide Experimental Datasets. Nucleic Acids Res (2019) 47(D1):D607–13. 10.1093/nar/gky1131 PMC6323986 30476243

[27] Subramanian A Tamayo P Mootha VK Mukherjee S Ebert BL Gillette MA. Gene Set Enrichment Analysis: A Knowledge-Based Approach for Interpreting Genome-Wide Expression Profiles. Proc Natl Acad Sci USA (2005) 102(43):15545–50. 10.1073/pnas.0506580102 PMC1239896 16199517

[28] Yoshihara K Shahmoradgoli M Martinez E Vegesna R Kim H Torres-Garcia W. Inferring Tumour Purity and Stromal and Immune Cell Admixture From Expression Data. Nat Commun (2013) 4:2612. 10.1038/ncomms3612 24113773 PMC3826632

[29] Chung SM Park JC Moon JS Lee JY. Novel Nomogram for Screening the Risk of Developing Diabetes in a Korean Population. Diabetes Res Clin Pract (2018) 142:286–93. 10.1016/j.diabres.2018.05.036 29885388

[30] Liu J Chen S Wang W Ning BF Chen F Shen W. Cancer-Associated Fibroblasts Promote Hepatocellular Carcinoma Metastasis Through Chemokine-Activated Hedgehog and TGF-beta Pathways. Cancer Lett (2016) 379(1):49–59. 10.1016/j.canlet.2016.05.022 27216982

[31] Riester M Taylor JM Feifer A Koppie T Rosenberg JE Downey RJ. Combination of a Novel Gene Expression Signature With a Clinical Nomogram Improves the Prediction of Survival in High-Risk Bladder Cancer. Clin Cancer Res (2012) 18(5):1323–33. 10.1158/1078-0432.CCR-11-2271 PMC3569085 22228636

[32] Peng M Wang Z Yang Z Tao L Liu Q Yi LU. Overexpression of Short TRPM8 Variant Alpha Promotes Cell Migration and Invasion, and Decreases Starvation-Induced Apoptosis in Prostate Cancer LNCaP Cells. Oncol Lett (2015) 10(3):1378–84. 10.3892/ol.2015.3373 PMC4533716 26622677

[33] Quan J Pan X Zhao L Li Z Dai K Yan F. LncRNA as a Diagnostic and Prognostic Biomarker in Bladder Cancer: A Systematic Review and Meta-Analysis. Onco Targets Ther (2018) 11:6415–24. 10.2147/OTT.S167853 PMC6177400 30323619

[34] Sanguedolce F Russo D Mancini V Selvaggio O Calo B Carrieri G. Prognostic and Therapeutic Role of HER2 Expression in Micropapillary Carcinoma of the Bladder. Mol Clin Oncol (2019) 10(2):205–13. 10.3892/mco.2018.1786 PMC6327213 30680196

[35] Sanguedolce F Calo B Mancini V Zanelli M Palicelli A Zizzo M. Non-Muscle Invasive Bladder Cancer With Variant Histology: Biological Features and Clinical Implications. Oncology (2021) 99(6):345–58. 10.1159/000514759 33735905

[36] Tilki D Burger M Dalbagni G Grossman HB Hakenberg OW Palou J. Urine Markers for Detection and Surveillance of non-Muscle-Invasive Bladder Cancer. Eur Urol (2011) 60(3):484–92. 10.1016/j.eururo.2011.05.053 21684071

[37] Liu A Zhang Z Xu W Qin S Hua M Zeng S. Overexpression of Long Noncoding RNA n346372 in Bladder Cancer Tissues is Associated With a Poor Prognosis. Mol Med Rep (2018) 18(6):5437–44. 10.3892/mmr.2018.9597 PMC6236288 30365104

[38] Wang C Tao W Ni S Chen Q. Upregulation of lncRNA Snorna Host Gene 6 Regulates NUAK Family SnF1-Like Kinase-1 Expression by Competitively Binding microRNA-125b and Interacting With Snail1/2 in Bladder Cancer. J Cell Biochem (2019) 120(1):357–67. 10.1002/jcb.27387 30168179

[39] Dai Q Li J Zhou K Liang T. Competing Endogenous RNA: A Novel Posttranscriptional Regulatory Dimension Associated With the Progression of Cancer. Oncol Lett (2015) 10(5):2683–90. 10.3892/ol.2015.3698 PMC4665929 26722227

[40] He RQ Huang ZG Li TY Wei YP Chen G Lin XG. RNA-Sequencing Data Reveal a Prognostic Four-LncRNA-Based Risk Score for Bladder Urothelial Carcinoma: An in Silico Update. Cell Physiol Biochem (2018) 50(4):1474–95. 10.1159/000494647 30359990

[41] Guo F Fu Q Wang Y Sui G. Long Non-Coding RNA NR2F1-AS1 Promoted Proliferation and Migration Yet Suppressed Apoptosis of Thyroid Cancer Cells Through Regulating miRNA-338-3p/CCND1 Axis. J Cell Mol Med (2019) 23(9):5907–19. 10.1111/jcmm.14386 PMC6714216 31304680

[42] Wang L Zhao S Mingxin YU. LncRNA NR2F1-AS1 Is Involved in the Progression of Endometrial Cancer by Sponging miR-363 to Target SOX4. Pharmazie (2019) 74(5):295–300. 10.1691/ph.2019.8905 31109400

[43] Huang H Chen J Ding CM Jin X Jia ZM Peng J. LncRNA NR2F1-AS1 Regulates Hepatocellular Carcinoma Oxaliplatin Resistance by Targeting ABCC1 Via miR-363. J Cell Mol Med (2018) 22(6):3238–45. 10.1111/jcmm.13605 PMC5980138 29602203

[44] Munger JS Sheppard D. Cross Talk Among TGF-Beta Signaling Pathways, Integrins, and the Extracellular Matrix. Cold Spring Harb Perspect Biol ( 2011) 3(11):a005017. 10.1101/cshperspect.a005017 21900405 PMC3220354

[45] Chakravarthy A Khan L Bensler NP Bose P De Carvalho DD. TGF-Beta-Associated Extracellular Matrix Genes Link Cancer-Associated Fibroblasts to Immune Evasion and Immunotherapy Failure. Nat Commun (2018) 9(1):4692. 10.1038/s41467-018-06654-8 30410077 PMC6224529

[46] Mariathasan S Turley SJ Nickles D Castiglioni A Yuen K Wang Y. Powles: TGFbeta Attenuates Tumour Response to PD-L1 Blockade by Contributing to Exclusion of T Cells. Nature (2018) 554(7693):544–8. 10.1038/nature25501 PMC6028240 29443960

[47] Zhang L Wu B Zha Z Qu W Zhao H Yuan J. Clinicopathological Factors in Bladder Cancer for Cancer-Specific Survival Outcomes Following Radical Cystectomy: A Systematic Review and Meta-Analysis. BMC Cancer (2019) 19(1):716. 10.1186/s12885-019-5924-6 31324162 PMC6642549

[48] Yao Z Zheng Z Ke W Wang R Mu X Sun F. Prognostic Nomogram for Bladder Cancer With Brain Metastases: A National Cancer Database Analysis. J Transl Med (2019) 17(1):411. 10.1186/s12967-019-2109-7 31815624 PMC6902467

[49] Mir MC Marchioni M Zargar H Zargar-Shoshtari K Fairey AS Mertens LS. Nomogram Predicting Bladder Cancer-Specific Mortality After Neoadjuvant Chemotherapy and Radical Cystectomy for Muscle-Invasive Bladder Cancer: Results of an International Consortium. Eur Urol Focus (2020). 10.1016/j.euf.2020.07.002 32771446

