# Supplementary material for: Integrated Analysis of a Competing Endogenous RNA Network Reveals a Prognostic lncRNA Signature in Bladder Cancer
Source: Front Oncol. 2021 Aug 2;11:684242. doi: 10.3389/fonc.2021.684242 (PMC8366562; doi:10.3389/fonc.2021.684242)
Supplement: Supplementary file 1 [file DataSheet_1.docx]

**Integrated analysis of a competing endogenous RNA network reveals a prognostic lncRNA signature in bladder cancer**

Mou Peng^#, *^, Xu Cheng^#^, Wei Xiong, Lu Yi, Yinhuai Wang^*^

Department of Urology, The Second Xiangya Hospital, Central South University, Changsha, 410011, Hunan, China.

^#^ These authors contributed equally to this work

*Corresponding Author

Department of Urology,

The Second Xiangya Hospital, Central South University

No.139 Renmin Middle Rd, Changsha, Hunan 410011, China

Tel: 86-731-85295134

Fax: 86-731-85295134

Email: Mou Peng: pengmou@csu.edu.cn&Yinhuai Wang: wangyinhuai@csu.edu.cn

| Group | Cases | Percent (%) |
| --- | --- | --- |
| Age |  |  |
| <60 | 87 | 21.59% |
| >=60 | 316 | 78.41% |
| Gender |  |  |
| Male | 297 | 73.70% |
| Female | 106 | 26.30% |
| Race |  |  |
| White | 320 | 79.40% |
| Asian | 43 | 10.67% |
| Black or African American | 23 | 5.71% |
| Pathologic stage |  |  |
| Stage I | 2 | 0.50% |
| Stage II | 129 | 32.01% |
| Stage III | 138 | 34.24% |
| Stage IV | 132 | 32.75% |
| Pathologic T |  |  |
| T1 | 3 | 0.74% |
| T2 | 118 | 29.28% |
| T3 | 191 | 47.39% |
| T4 | 57 | 14.14% |
| Pathologic M |  |  |
| M0 | 194 | 48.14% |
| M1 | 11 | 2.73% |
| Mx | 195 | 48.39% |
| Pathologic N |  |  |
| N0 | 234 | 58.06% |
| N1 | 45 | 11.17% |
| N2 | 75 | 18.61% |
| N3 | 7 | 1.74% |
| Nx | 36 | 8.93% |
| Vital status |  |  |
| Alive | 226 | 56.08% |
| Dead | 177 | 43.92% |

Supplementary Table 1 Clinicopathological characteristics of 403 bladder cancer patients

| Category | Term | PValue | Genes |
| --- | --- | --- | --- |
| GOTERM_BP_DIRECT | GO:0000122 negative regulation of transcription from RNA polymerase II promoter | 0.001 | HOXB3, EGR1, FGFR1, HDAC4, MITF, ZFPM2, RARB, DNAJB5 |
| GOTERM_BP_DIRECT | GO:0051216 cartilage development | 0.009 | CSGALNACT1, HOXB3, ZEB1 |
| GOTERM_BP_DIRECT | GO:0010863 positive regulation of phospholipase C activity | 0.018 | FGFR1, PDGFRA |
| GOTERM_BP_DIRECT | GO:0006351 transcription, DNA-templated | 0.021 | CEBPA, EGR1, HDAC4, THRA, POU2F2, RARB |
| GOTERM_BP_DIRECT | GO:0030324 lung development | 0.022 | CTGF, PDGFRA, ZFPM2 |
| GOTERM_BP_DIRECT | GO:0030198 extracellular matrix organization | 0.032 | RECK, CSGALNACT1, PDGFRA |
| GOTERM_BP_DIRECT | GO:0007229 integrin-mediated signaling pathway | 0.041 | CTGF, ITGA5, ADAM12 |
| GOTERM_BP_DIRECT | GO:0098792 xenophagy | 0.046 | PDK4, MYLK, FAM13B |
| GOTERM_CC_DIRECT | GO:0005634 nucleus | 0.028 | EGR1, THRA, MITF, ZEB1, PALLD, CCNL2, HOXB3, POU2F2, RNF38, PDGFRA, CELF2, RARB, PBX3, DNAJB5 |
| GOTERM_MF_DIRECT | GO:0043565 sequence-specific DNA binding | 0.003 | CEBPA, HOXB3, HDAC4, THRA, POU2F2, RARB, PBX3 |
| GOTERM_MF_DIRECT | GO:0003700 DNA-binding transcription factor activity | 0.022 | CEBPA, HOXB3, MITF, POU2F2, RUNX1T1, PBX3 |
| GOTERM_MF_DIRECT | GO:0003714 transcription corepressor activity | 0.048 | HDAC4, RUNX1T1, ZFPM2 |
| KEGG_PATHWAY | cjc05206: MicroRNAs in cancer | 0.000 | RECK, ERBB3, ITGA5, PDGFRA, ZFPM2, ZEB1, PLAU |
| KEGG_PATHWAY | cjc04810: Regulation of actin cytoskeleton | 0.012 | FGFR1, ITGA5, CFL2, PDGFRA, MYLK |
| KEGG_PATHWAY | cjc05200: Pathways in cancer | 0.018 | CEBPA, FGFR1, MITF, PDGFRA, RUNX1T1, RARB |
| KEGG_PATHWAY | cjc05202: Transcriptional misregulation in cancer | 0.031 | CEBPA, RUNX1T1, PBX3, PLAU |
| KEGG_PATHWAY | cjc05218: Melanoma | 0.033 | FGFR1, MITF, PDGFRA |

Supplementary Table 2 GO and KEGG pathways of mRNA involved in ceRNA network

| lncRNA | HR | Z score | P value |
| --- | --- | --- | --- |
| AC010168.2 | 0.729065 | -4.75124 | 2.02E-06 |
| RNF139-AS1 | 0.692169 | -3.90447 | 9.44E-05 |
| RUSC1-AS1 | 0.715231 | -3.7573 | 0.00017 |
| NR2F1-AS1 | 1.209124 | 3.520819 | 0.00043 |
| LINC01355 | 0.784443 | -3.51076 | 0.00045 |
| ARHGAP27P1-BPTFP1-KPNA2P3 | 0.763355 | -3.4971 | 0.00047 |
| CAPN10-AS1 | 0.718915 | -3.42363 | 0.00062 |
| AC084125.2 | 0.825342 | -3.38376 | 0.00072 |
| LINC01311 | 0.764463 | -3.37331 | 0.00074 |
| C17orf82 | 0.852143 | -3.35631 | 0.00079 |
| AC074117.1 | 0.726767 | -3.13447 | 0.00172 |
| LINC01341 | 0.902694 | -3.12335 | 0.00179 |
| SNHG10 | 0.730691 | -3.09527 | 0.00197 |
| MIR100HG | 1.122259 | 2.999377 | 0.00271 |
| AC008105.1 | 0.829833 | -2.97554 | 0.00292 |
| PDXDC2P-NPIPB14P | 0.803416 | -2.94403 | 0.00324 |
| AC027601.1 | 0.779424 | -2.85961 | 0.00424 |
| AC105942.1 | 1.188236 | 2.808018 | 0.00498 |
| LINC01481 | 0.801365 | -2.5624 | 0.0104 |
| AC008105.2 | 0.877397 | -2.53612 | 0.01121 |
| SNHG1 | 0.781433 | -2.42 | 0.01552 |
| SNHG12 | 0.781433 | -2.42 | 0.01552 |
| AC074212.1 | 0.844367 | -2.31739 | 0.02048 |
| MIR4435-2HG | 1.139647 | 2.23053 | 0.02571 |
| PCAT7 | 0.915398 | -2.21898 | 0.02649 |
| DNM3OS | 1.101377 | 2.048545 | 0.04051 |
| AP001207.3 | 0.941787 | -1.97119 | 0.0487 |

Supplementary Table 3 Prognostic lncRNAs in ceRNA network using univariate Cox analysis

Supplementary Table 4 Prognostic lncRNAs in ceRNA network using multivariate Cox analysis

| id | coef | exp(coef) | se(coef) | z | Pr(>\|z\|) |
| --- | --- | --- | --- | --- | --- |
| AC010168.2 | -0.2863566 | 0.7509948 | 0.06775174 | -4.226556 | 2.37E-05 |
| NR2F1-AS1 | 0.1498769 | 1.1616912 | 0.05476237 | 2.736859 | 6.20E-03 |

Supplementary Figure 1


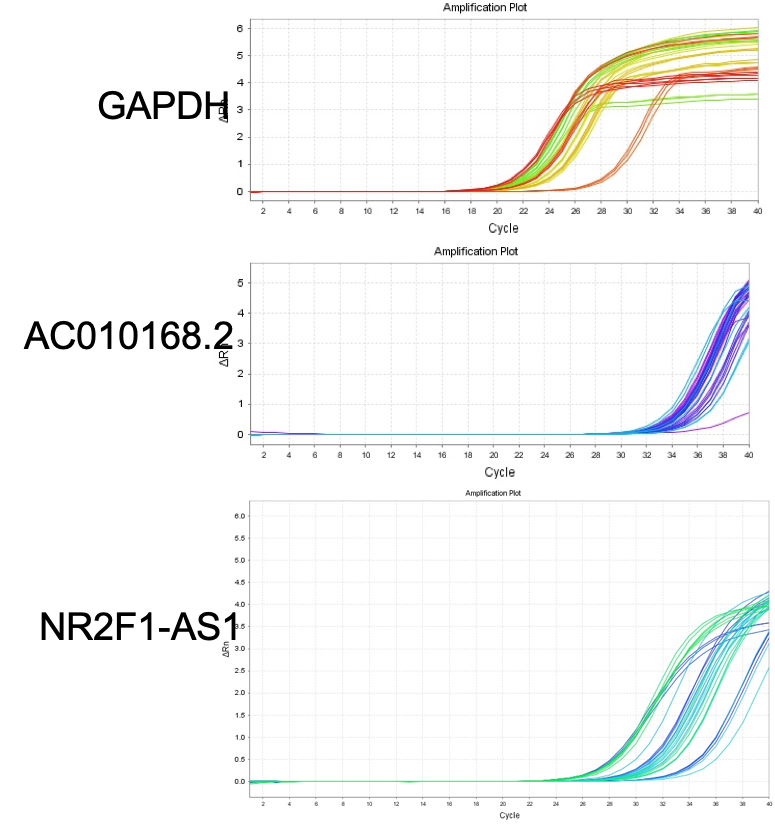


Amplification curves of PCR

Supplementary Figure 2


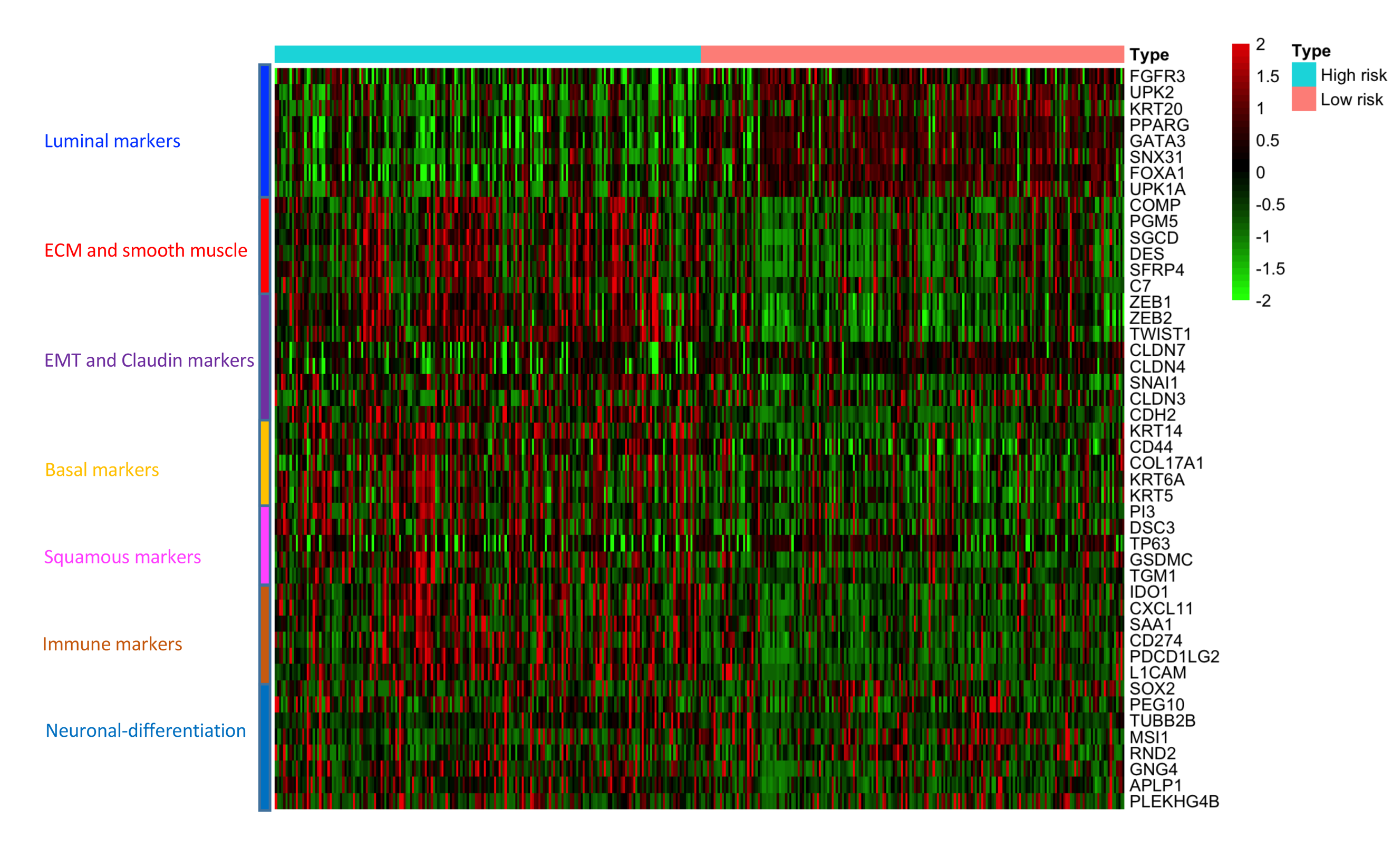


Heatmap of characterized signatures in TCGA-BLCA cohort.
